# Bioinformatic Identification and Expression Profiling of Heptahelical Transmembrane Protein Genes in Soybean Under Phytohormone and Nematode Stress

**DOI:** 10.3390/biology14091223

**Published:** 2025-09-09

**Authors:** Wenshu Kang, Nawei Qi, Piao Lei

**Affiliations:** 1 Key Laboratory of Ecological Restoration of Regional Contaminated Environment, Ministry of Education, College of Environment, Shenyang University, Shenyang 110044, China; kangwenshu@126.com; 2 College of Life Science, Shenyang Normal University, Shenyang 110034, China; nwqi199208@163.com; 3 State Key Laboratory of Natural Medicines, China Pharmaceutical University, Nanjing 211198, China; 4 Institut Sophia Agrobiotech, 06903 Sophia Antipolis, France

**Keywords:** heptahelical transmembrane protein genes, soybean, phytohormone, cyst nematode

## Abstract

Heptahelical transmembrane proteins (HHPs) have recently been recognized as crucial regulators of diverse biological processes in eukaryotes. In this study, 10 *GmHHP* genes were identified in soybean, and a comprehensive analysis was conducted to examine their phylogenetic relationships, cis-regulatory elements, expression patterns, and potential regulatory networks. Expression profiling revealed that most *GmHHP* genes were transcriptionally induced by abscisic acid (ABA) and methyl jasmonate (MeJA), with *GmHHP1* and *GmHHP7* exhibiting the strongest induction. During soybean cyst nematode (SCN) infection, several *GmHHP* genes were down-regulated, suggesting a potential role in plant–nematode interactions. Protein interaction network analysis indicated that *GmHHPs* could interact with mitochondrial pyruvate carriers, alkaline phytoceramidases, and histone deacetylases, which may link them to ABA-regulated biological processes such as stomatal movement, water homeostasis, and stress adaptation. Furthermore, interacting miRNA prediction demonstrated that conserved miRNAs, including miR172 and miR319, might co-regulate *GmHHP* genes and their associated protein partners. Collectively, these findings indicate that *GmHHP* genes function as membrane-associated regulators of ABA signaling and defense responses, particularly under biotic stress, such as nematode infection.

## Simple Summary

Heptahelical transmembrane proteins have recently been recognized as crucial regulators of diverse biological processes. In this study, we identified ten genes encoding heptahelical transmembrane proteins in soybean. Expression profiling showed that these genes were transcriptionally regulated by abscisic acid, methyl jasmonate, and soybean cyst nematode infection. Protein interaction network analysis and microRNA prediction suggested that these genes may interact with other proteins and conserved microRNAs involved in phytohormone pathways and stress responses.

## 1. Introduction

Plants inhibit environments continuously exposed to diverse stimuli and challenges. To survive, they have evolved strategies to perceive external conditions and respond to both abiotic and biotic stresses [1]. These strategies involve either direct sensing mechanisms or molecular interaction-based pathways. At the molecular level, a wide range of receptor genes, including photoreceptors, phytohormone receptors, and pattern recognition receptors (PRRs), facilitate the perception of environmental factors such as light, phytohormones, and biotic stresses [2,3,4]. To counter pathogen attack, nucleotide-binding leucine-rich repeat receptor (NLR) and PRR genes function in effector-triggered immunity (ETI) and pattern-triggered immunity (PTI), respectively [5]. PRRs are located in the plasma membrane, where they detect external signals including pathogen-associated molecular patterns (PAMPs), damage-associated molecular patterns (DAMPs), microbe-associated molecular patterns (MAMPs), and herbivore-associated molecular patterns (HAMPs), as well as stress-induced phytocytokines, to trigger immune responses [4].

The human progestin and adipoQ receptor (PAQR) gene family encodes a group of membrane proteins, including receptors for steroid hormones and adiponectin, which participate in various physiological processes such as metabolism, reproduction, and stress responses [6,7]. This receptor family, mainly composed of adiponectin receptors (AdipoRs) and membrane progestin receptors (mPRs), interacts with intracellular signaling components, such as G proteins, AMP-activated protein kinase (AMPK), and mitogen-activated protein kinase/extracellular signal-regulated kinases (MAPK/ERKs) to regulate diverse biological processes [6].

In plants, homologs of human PAQRs have been annotated as heptahelical transmembrane proteins (HHPs) in multiple species (https://phytozome-next.jgi.doe.gov/, accessed on 5 March 2025). However, their biological roles remain largely unknown. Notably, their potential interacting partners, G proteins and AMPK, play roles in various biological pathways. Plant G proteins are heterotrimeric complexes consisting of α, β, and γ subunits that regulate hormone signaling, development, stress adaption, and immune response [8]. In Arabidopsis, the AMPK ortholog SNF1-related kinase 1 (SnRK1) serves as a central regulator of energy metabolism, particularly under energy-deficient conditions such as starvation, darkness, or stress. For example, the Arabidopsis transcription factor bZIP63 is a direct target of SnRK1. The phosphorylation of bZIP63 by SnRK1 alters its dimerization, thereby modulating target gene expression and influencing primary metabolism [9,10]. These observations suggest *HHP* genes, as a class of receptor-like genes, may coordinate plant immunity and responses to environmental stimuli. In Arabidopsis, Hiseh and Goodman identified the *AtHHP* gene family, which is structurally similar to AdipoRs and mPRs. The expression of *AtHHP* genes can be differentially regulated by plant hormones, sucrose, temperature, and salt stress, implying that these conserved transmembrane proteins may function as a new class of receptors [11,12].

*In silico* and *in vivo* analyses of plant genomic datasets have provided valuable insights into the functions of plant receptor genes and their molecular mechanisms, thereby deepening our understanding, and facilitating the enhancement, of broad-spectrum disease resistance in crops, contributing to sustainable agricultural practices [13]. Soybean is a crucial source of protein and oil for humans. However, global infestations of soybean parasitic nematodes, particularly the soybean cyst nematode (SCN), have caused yield losses of 10% to 15% and resulted in estimated annual economic losses of nearly USD 78 billion [14]. Currently, identification of SCN-resistance genes remains a primary strategy for effective control [15]. Based on the demonstrated role of *HHP* receptor genes in enhancing disease resistance in other plants, this study conducted a comprehensive analysis of the *HHP* gene family in soybean, encompassing gene structure, conserved motifs, phylogenetic relationships, gene synteny, and expression patterns under phytohormone treatments and SCN infections. In addition, we investigated the miRNAs regulating these genes, and a regulatory network of their interacting proteins was constructed. This study provides new insights and establishes a theoretical basis for functional research on soybean *HHP* genes and disease-resistance breeding.

## 2. Materials and Methods

### 2.1. Identification of HHP Genes in Soybean Genome

The genome sequences, protein sequences, and GFF3 annotation files of *G. max* (Wm82.a4.v1), *A. thaliana* (TAIR10), and *G. soja* (Gsoja_509_v1.0) were downloaded from the Phytozome database (https://phytozome-next.jgi.doe.gov/, accessed on 4 April 2025). The protein sequences files were used to build a local BLAST+ 2.17.0 database using TBtools software (V2.310) [16]. Then, the Hidden Markov Model (HMM) profile corresponding to PF03006 was utilized to query this database through BLASTP with an *E*-value threshold of <1 × 10^−5^. In parallel, amino acid sequences of AtHHPs were employed as queries in separate BLASTP analyses against the *G. max* dataset using a threshold of <1 × 10^−5^. The combined results from both strategies were used to further analysis.

### 2.2. Conserved Domain, Motif Identification, Gene Structure, and Chromosomal Distribution Analysis

We used the sequences candidate *HHP* genes identified by HMM search and BLAST to query Conserved Domain Database (CDD). The presence of the PF03006 domain was confirmed using the CD-Search tools provided by NCBI (https://www.ncbi.nlm.nih.gov/cdd/, accessed on 5 April 2025). Specifically, a CDD search was performed against the CDD-62456 PSSM database with an *E*-value threshold of <0.01, retrieving up to 500 hits. Conserved motifs were predicted using the MEME Suite (https://meme-suite.org/meme/tools/meme, accessed on 5 April 2025), with the maximum number of motifs set to three and all other parameters kept at default. Genes lacking the PF03006 domain and any homologous conserved motifs were removed from further analysis. The gene structures of validated *HHP* genes from *G. max* were visualized using TBtools based on the GFF3 annotation file. These genes were localized to specific chromosomes in the soybean genome.

### 2.3. Phylogenetic Evolution, Physicochemical Properties, and Subcellular Localization of GmHHPs

Protein sequences of *GmHHP* genes and their homologs in Arabidopsis were aligned using ClustalW implemented in MEGA X, with default parameters (gap opening penalty = 10, gap extension penalty = 0.2). A neighbor-joining (NJ) phylogenetic tree was subsequently constructed based on the Poisson correction model, with gaps treated by pairwise deletion. The reliability of each branch was assessed by bootstrap analysis with 1000 replicates [17]. The ProtParam tool (https://web.expasy.org/protparam/, accessed on 5 April 2025) was employed to analyze physicochemical characteristics such as amino acid composition, molecular mass, and theoretical pI values. Subcellular localization of GmHHP was predicted using Plant-mPLoc (http://www.csbio.sjtu.edu.cn/bioinf/Cell-PLoc-2, accessed on 5 April 2025).

### 2.4. Duplication and Collinearity Analysis of HHP Genes

Tandem and segmental duplications of *GmHHP* genes were identified using the MCScanX toolkit with default settings [18]. The Dual Synteny Plotter module was applied to further examine tandem duplication and orthologous relationships of *HHP* genes between soybean and two additional species *(A. thaliana* and *G. soja*).

### 2.5. Prediction of cis-Elements, Interacting Proteins, and Regulating miRNA

To identify *cis*-regulatory elements, 2000 bp upstream sequences of the GmHHP- and AtHHP-coding regions were retrieved from genome annotations based on GFF3 files. These promoter regions were subsequently analyzed using the PlantCARE database (http://plantpan.itps.ncku.edu.tw/index.html, accessed on 5 April 2025). The protein sequences of GmHHP were submitted to the STRING database (https://string-db.org/, accessed on 10 April 2025) to predict potential protein–protein interactions. Enrichment analysis of the biological processes regulated by the interacting proteins was subsequently conducted. Furthermore, the CDS of *GmHHP* and its predicted interactors were uploaded to the psRNATarget server (https://www.zhaolab.org/psRNATarget/, accessed on 17 April 2025) for miRNA target prediction, using the default parameters of schema V2 (2017 release), with a maximum expectation value of 3.0.

### 2.6. Plant Material and Phytohormone Treatments

Soybean (Williams 82) seeds were surface-sterilized with 1% sodium hypochlorite (NaClO) for 10 min and then thoroughly rinsed with sterile distilled water. The sterilized seeds were germinated in vermiculite moistened with Hoagland nutrient solution at a photoperiod 16/8 (light/dark) with 25 °C in a greenhouse. Five seven-day-old soybean seedlings with fully expanded primary leaves were transferred to ABA and MeJA treatment solutions for 12 h with three biological replicates. Both ABA and MeJA were applied at concentrations of 0, 10, 40, and 160 μM, respectively.

### 2.7. GmHHP Gene Expression Analysis Under Nematode Infection

Transcriptome data of soybean under nematode infection were downloaded from the studies by Kang et al. [19]. and Qi et al. [20]. Based on these datasets, the expression profiles of *GmHHP* genes were extracted, and a heatmap was generated using the log_2_(fold change) values of *GmHHPs*. SCN was used as biotic stress to test the response of *GmHHP* genes. Briefly, freshly hatched second-stage juveniles (J2s) of SCN race 3 (HG type 0) were inoculated to the ten-day-old soybean (Williams 82) roots. Five non-infected and SCN-infected soybean roots were collected at 48 h post-inoculation (hpi) and 8 dpi with three biological replicates.

### 2.8. RNA Extraction and qRT-PCR Analysis

Total RNA of soybean roots was isolated using the Ultrapure RNA Kit (CWbiotech, Beijing, China) following the manufacturer’s instructions. RNA concentration and purity were measured with a NanoDrop 2000 spectrophotometer (Thermo Fisher Scientific, Wilmington, DE, USA). First-strand cDNA was synthesized from 1 μg of total RNA using the SYBR PrimeScript RT Master Mix kit (Takara, Dalian, China). Quantitative real-time PCR (qRT-PCR) was performed on a CFX Connect Real-Time PCR Detection System (Bio-Rad, Hercules, CA, USA) under the following cycling conditions: 95 °C for 2 min, followed by 40 cycles of 95 °C for 5 s and 60 °C for 30 s. The soybean *60SRP* gene was used as the internal reference. Relative expression levels were calculated using the 2^^−ΔΔCt^ method [21]. Each qRT-PCR analysis included three biological replicates and three technical replicates.

## 3. Results

### 3.1. Identification of GmHHP Genes in Soybean Genome

**Figure 1:**
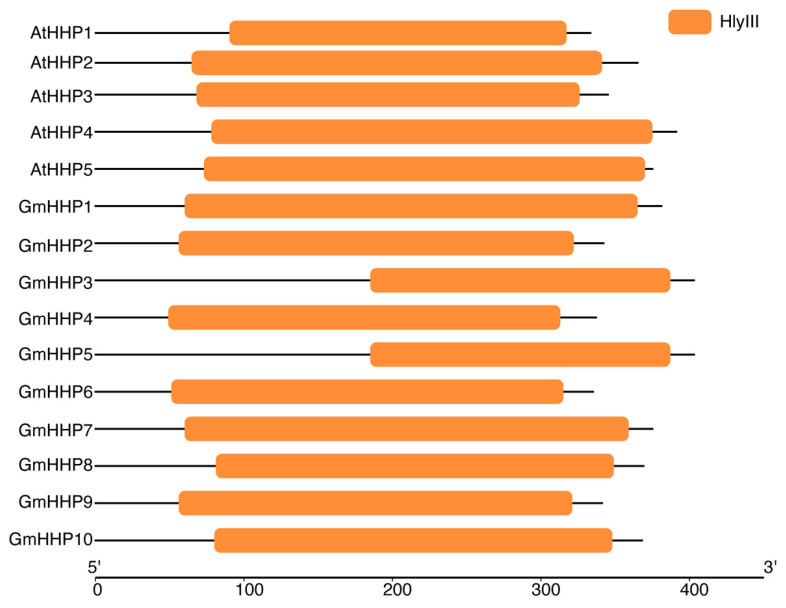
Conserved domain identification of AtHHPs and GmHHPs. The conserved domains of HHPs from *A. thaliana* and *Glycine max* were identified using NCBI CDD. All identified HHPs contained domains belong to the Hemolysin-III related (HlyIII) family. Colored boxes represent conserved domains mapped along amino acid sequences. The x-axis indicates the protein length from the N-terminus (5′) to the C-terminus (3′).

To identify soybean heptahelical transmembrane protein-coding genes (*GmHHPs*)*,* the HMM profile of PF03006 was retrieved and used to query a local soybean protein database, which identified 23 putative *GmHHP* genes (Table S1). Moreover, the protein sequences of five *AtHHP* genes (from *Arabidopsis thaliana*) were obtained from the TAIR database and used as queries in a BLAST search against the soybean protein datasets, leading to the identification of 10 candidate *GmHHP* genes (Table S1). The protein sequences of all genes identified by these two methods were submitted to the Conserved Domain Database (CDD) to confirm the presence of Hemolysin-III related (HlyIII) conserved domains. Five *AtHHP* genes (AT5G20270, AT4G30850, AT2G24150, AT4G37680, and AT4G38320) were found to contain the HlyIII domain. Similarly, this conserved domain was also present in 10 candidate *GmHHP* genes (Glyma.01G194900, Glyma.02G207000, Glyma.04G046500, Glyma.04G156100, Glyma.06G047200, Glyma.06G223600, Glyma.11G046900, Glyma.13G161500, Glyma.17G070900, and Glyma.17G109800) (Figure 1 and Table S1). Therefore, the remaining 13 genes were excluded from further analysis. The validated soybean genes were designated as *GmHHP1* through *GmHHP10* (Figure 1 and Table S1).

**Figure 2:**
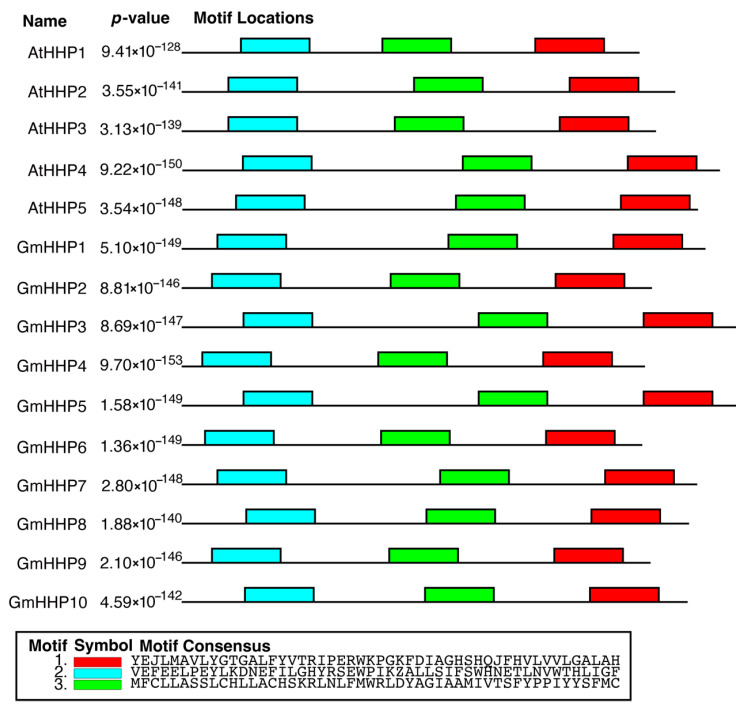
Conserved motif analysis of HHPs from *A. thaliana* and *G. max*. The conserved motifs within HHPs were identified using the MEME Suite. The schematic diagram shows the distribution of three conserved motifs across individual GmHHP sequences. Each colored box represents a distinct motif positioned along the protein from the N-terminus (5′) to the C-terminus (3′).

Further analysis using MEME revealed that *HHP* genes from both *A. thaliana* and soybean shared three conserved motif structures (Figure 2). Each motif consisted of approximately 50 amino acid residues.

To investigate the physicochemical properties of soybean HHPs, the amino acid sequences of *GmHHP* were analyzed using the ProtParam tool (http://web.expasy.org/protparam/, accessed on 5 April 2025). The lengths of GmHHPs ranged from 334 to 402 amino acid residues, with molecular weights between 37,957 and 46,245 Da, as well as theoretical isoelectric points (pI) ranging from 8.04 to 9.24. Subcellular localization prediction with Plant-mPLoc indicated that all GmHHPs could be localized to the plasma membrane (Table 1).

### 3.2. Gene Structure, Chromosomal Distribution, and Phylogenetic Analysis

**Figure 3:**
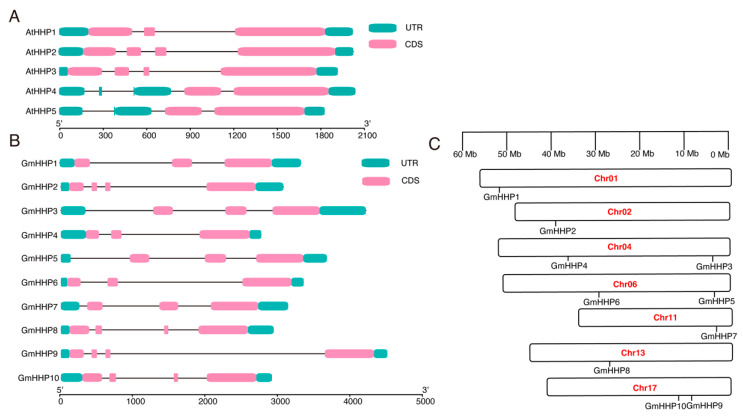
Structure and chromosomal distribution of *HHP* genes in soybean. (**A**) Structure of *AtHHP* genes. (**B**) Structure of *GmHHP* genes. Untranslated regions (UTRs) are marked with green boxes, while coding sequences (CDS) are shown as purple or pink boxes. Introns are represented by black lines. (**C**) Chromosomal localization of *GmHHP* genes on soybean chromosomes Gm01, Gm04, Gm06, Gm11, Gm12, Gm13, and Gm17. The x-axis indicates gene length from the 5′ to 3′ end.

To elucidate the structural composition of *GmHHP* genes, a gene structure map was generated from the soybean genome sequence, highlighting the untranslated regions (UTRs), coding sequences (CDSs), and introns (Figure 3). Structural variations were observed in *GmHHP* genes and their homologs in *A. thaliana*. For example, in Arabidopsis, *AtHHP1* contains three CDSs, *AtHHP2* and *AtHHP3* each contain four CDSs, and *AtHHP4* and *AtHHP5* possess two CDSs. In soybean, 3 of the 10 *GmHHP* genes (*GmHHP2*, *GmHHP8*, and *GmHHP9*) also contained four CDSs. Differences were also observed in the number of UTRs. Except for *AtHHP4* and *AtHHP5*, which contain more than three UTRs, all other *HHP* genes in both soybean and Arabidopsis possessed only three UTRs (Figure 3A,B). The 10 *GmHHP* genes were unevenly distributed across seven soybean chromosomes. *GmHHP1* was located on chromosome 1 (Chr01), *GmHHP2* on Chr02, *GmHHP3* and *GmHHP4* on Chr04, *GmHHP5* and *GmHHP6* on Chr06, *GmHHP7* and *GmHHP8* on Chr11 and Chr13, respectively, and *GmHHP9* and *GmHHP10* on Chr17 (Figure 3C).

**Figure 4:**
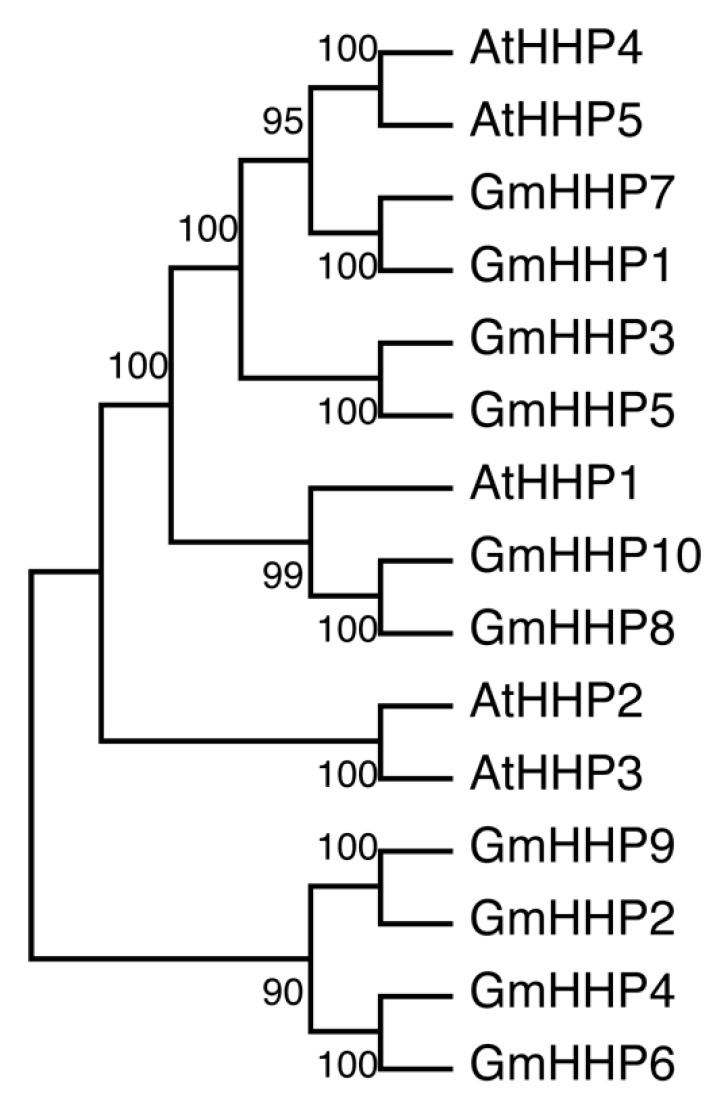
Phylogenetic analysis of GmHHPs.The phylogenetic tree was constructed based on the amino acid sequences of five AtHHPs proteins and ten GmHHPs, using the neighbor-joining (NJ) method implemented in MEGA11. Multiple sequence alignment was performed with ClustalW. Bootstrap analysis was conducted with 1000 replicates, and bootstrap values above branches indicate statistical support, suggesting evolutionary divergence among the HHP family members in Arabidopsis and soybean.

A phylogenetic tree was constructed using MEGA software (bootstrap value of 1000) based on the amino acid sequences of the ten identified GmHHPs, together with five AtHHPs. Phylogenetic analysis revealed that GmHHP1 and GmHHP7 clustered more closely with AtHHP4 and AtHHP5. GmHHP3, GmHHP5, GmHHP8, and GmHHP10 showed a closer evolutionary relationship with AtHHP1, whereas GmHHP2, GmHHP4, GmHHP6, and GmHHP9 were more closely related to AtHHP2 and AtHHP3 (Figure 4).

### 3.3. Gene Duplication and Collinearity

**Figure 5:**
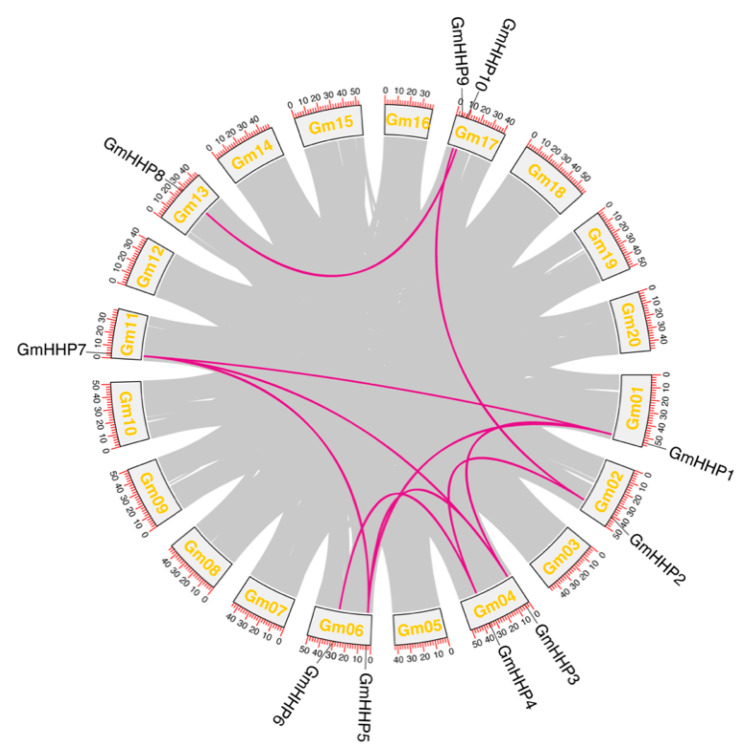
Gene duplication and collinearity analysis of *GmHHP* genes in the soybean genome. Genome-wide synteny analysis was performed to identify duplicated *GmHHP* gene pairs in *G. max*. The gray lines represent all duplicated gene pairs across the genome, while the purple lines highlight the collinear pairs within the *GmHHP* family. Chromosome numbers are displayed at the bottom of each chromosome, and renamed *GmHHP* gene IDs are presented above their corresponding loci.

A total of 10 segmental duplication events involving *GmHHP* genes were identified in the soybean genome, while no tandem duplications were detected (Figure 5 and Table S2). This analysis demonstrated that several *GmHHP* genes participated in duplication events across multiple chromosomes. For instance, *GmHHP1* was segmentally duplicated with *GmHHP3*, *GmHHP5*, and *GmHHP7*. Similarly, *GmHHP2* formed duplication pairs with both *GmHHP4* and *GmHHP9*. *GmHHP3* also shared duplication events with *GmHHP5* and *GmHHP7*, while *GmHHP5* was duplicated with *GmHHP7*. A duplicate relationship was also observed between *GmHHP4* and *GmHHP6*. Furthermore, *GmHHP8* and *GmHHP10* constituted another duplication pair (Figure 5 and Table S2).

**Figure 6:**
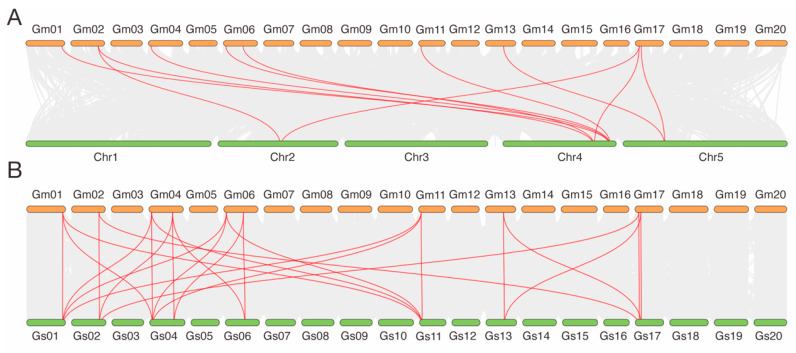
Collinearity analysis of *HHP* genes among *A. thaliana*, *G. max*, and *G. soja*. (**A**) Synteny analysis was performed to explore the evolutionary relationships of *HHP* genes among *A. thaliana* and *G. max*. (**B**) Synteny analysis of evolutionary relationships of *HHP* genes among *G. max* and *G. soja*. Gray lines indicate all syntenic blocks across the genomes, whereas red lines highlight collinear gene pairs specifically within the *HHP* gene family. Chromosomes from each species are labeled accordingly.

Collinearity analysis between soybean and *A. thaliana* revealed several orthologous relationships among *HHP* genes, indicating evolutionary conservation across species. Specifically, *GmHHP1* was collinear with *AtHHP2* and *AtHHP3*. *GmHHP2*, *GmHHP3*, *GmHHP5*, and *GmHHP7* were all collinear with *AtHHP3*. *GmHHP6* and *GmHHP9* were associated with *AtHHP2*. *GmHHP9* showing the collinearity with *AtHHP3* (Figure 6A and Table S2). Furthermore, *GmHHP8* and *GmHHP10* were collinear with *AtHHP1*. Further comparison with *Glycine soja* demonstrated 10 *GmHHP* genes had orthologous counterparts in the *Glycine soja* genome, reflecting strong evolutionary conservation between the cultivated and wild species. For example, *GmHHP1*, *GmHHP5*, and *GmHHP7* were all collinear with GlysoPI483463.01G156600, 04G043300, and 11G042500, respectively, while *GmHHP8* and *GmHHP10* corresponded to GlysoPI483463.13G127800 and 17G101600 (Figure 6B and Table S2).

### 3.4. cis-Regulating Element Analysis and Phytohormone Response of GmHHP Genes

To characterize the regulatory region of *GmHHP* promoters, approximately 2000 bp upstream sequences were analyzed. Sixteen categories of cis-acting elements were identified, including both *GmHHP* and *AtHHP* gene promoters, covering hormone responsiveness, hormonal responses, stress signaling, developmental regulation, and secondary metabolism. Light-responsive elements were particularly abundant, with *GmHHP4* and *GmHHP5* containing 13 and 12 such elements, respectively. Stress-related motifs, including those responsive to drought and low temperature, were also widely distributed. For example, *GmHHP7* contained seven low-temperature–responsive elements. Elements related to flavonoid biosynthesis were identified in *GmHHP3*, *GmHHP6*, and *GmHHP10*, suggesting their involvement in secondary metabolism (Table S3).

**Figure 7:**
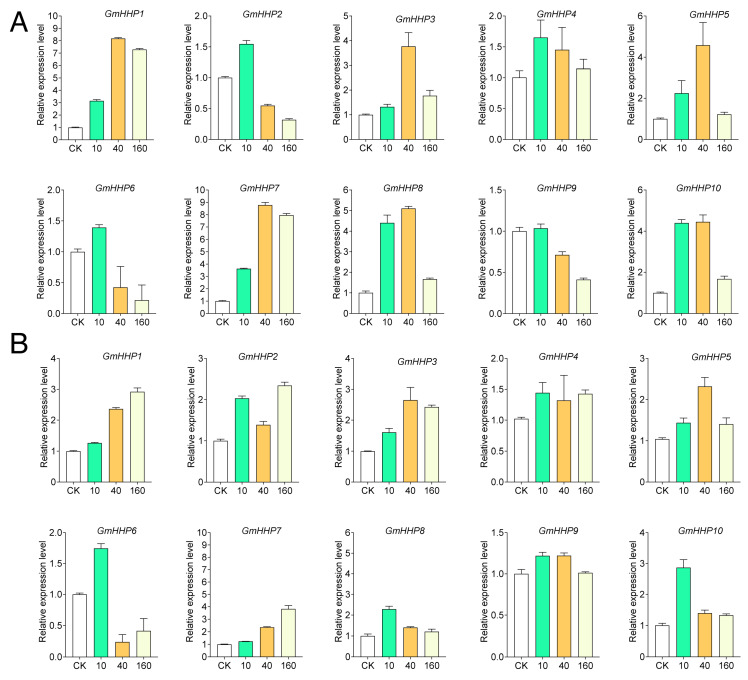
Expression patterns of *GmHHP* genes in response to ABA and MeJA treatments. (**A**) Expression profiles of *GmHHP* genes after treatment with a series of ABA concentrations (0, 10, 40, and 160 μM). (**B**) Expression profiles of GmHHP genes under various MeJA concentrations (0, 10, 40, and 160 μM). Soybean seedlings were treated for 12 h, and root tissues were collected for qRT-PCR analysis with three biological and three technical replicates.

Hormone-responsive elements are particularly prominent. All *GmHHP* genes contained abscisic acid (ABA)-responsive motifs, with *GmHHP8* carrying the highest number. MeJA-responsive elements were found in approximately 60% of the genes, implying the potential participation of this family in multiple hormone signaling pathways (Table S3). To validate these predictions, soybean roots were treated with different concentrations of ABA and MeJA, and the expression pattern profiles of *GmHHP* genes were subsequently analyzed. *GmHHP* genes exhibited differential expression under various concentrations of ABA and MeJA. Distinct responses were observed for both treatments (Figure 7). Under ABA treatment exposure, most *GmHHP* genes were up-regulated, particularly at 10 and 40 μM, except for *GmHHP2* and *GmHHP6*, which showed no significant induction (Figure 7A). In contrast, MeJA generally elicited a generally weaker response. Notably, *GmHHP6* was repressed at 40 and 160 μM MeJA, whereas the remaining genes were activated at all tested concentrations (Figure 7B). Some genes exhibited consistent patterns in both treatments. For instance, *GmHHP1* was strongly up-regulated at 40 and 160 μM ABA and MeJA, whereas *GmHHP6* was induced at 10 and 40 μM of both hormones but repressed at 160 μM (Figure 7).

### 3.5. GmHHP Genes Were Responsive to Nematode Infection

**Figure 8:**
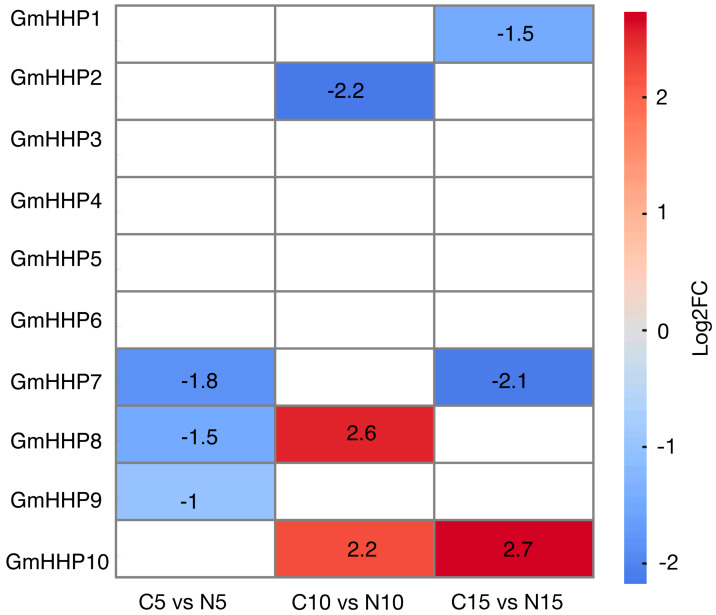
Expression profiles of *GmHHP* genes under SCN infection at different time points by using RNA-seq data. The heatmap illustrates the expression patterns of *GmHHP* genes in *G. max* based on RNA-seq data from Kang et al. and Qi et al. Expression values were normalized and log_2_-transformed (log_2_FC) to highlight relative changes across different samples or treatments. The color scale represents expression levels, where red indicates up-regulation and blue indicates down-regulation.

To determine whether *GmHHP* genes could respond to soybean cyst nematode (SCN) infection, we first analyzed publicly available transcriptome datasets. A heatmap was generated to visualize the expression profiles of *GmHHP* genes in soybean roots at 5, 10, and 15 days post-inoculation (dpi) with SCN. The results demonstrated that several *GmHHP* genes exhibited dynamic and time-specific responses to nematode infection. For instance, *GmHHP10* was markedly up-regulated, with log_2_(fold change) values of 2.2 and 2.7 observed at 10 and 15 dpi, respectively (Figure 8). Then, we also used qRT-PCR to detect the expression patterns of other *GmHHP* genes under SCN infection.

**Figure 9:**
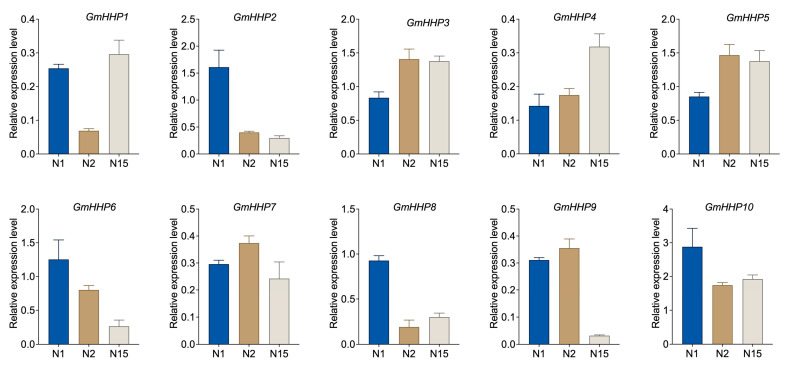
Expression patterns of *GmHHP* genes under SCN infection. Soybean seedlings were inoculated with 2000 second-stage juveniles (J2) of SCN. Roots from inoculated and non-inoculated (control) and SCN-inoculated plants were collected at 1, 2, and 15 dpi, corresponding to C1, C2, and C15 (control), as well as N1, N2, and N15 (infected), respectively. The gene expression levels of *GmHHP* genes were normalized to those of non-inoculated soybean roots. qRT-PCR analysis was conducted using three biological and three technical replicates.

To investigate the transcriptional response of *GmHHP* genes to SCN infection, qRT-PCR was performed to assess their expression in soybean roots at 1, 2, and 15 dpi. Notably, *GmHHP3*, *GmHHP4*, and *GmHHP5* were significantly up-regulated at both 2 and 15 dpi, suggesting their potential role in the sustained response to nematode invasion. *GmHHP1* exhibited a transient down-regulation at 2 dpi, followed by an increase at 15 dpi. In contrast, *GmHHP2* and *GmHHP6* demonstrated consistent down-regulation across all time points, suggesting suppression under nematode stress. *GmHHP10* displayed pronounced down-regulation at 2 dpi, with a slight recovery at 15 dpi. Other members, including *GmHHP7*, *GmHHP8*, and *GmHHP9*, displayed moderate or variable expression changes, with *GmHHP8* showing a distinct suppression at 2 dpi (Figure 9).

### 3.6. GmHHP Genes Influence Several Biological Processes

**Figure 10:**
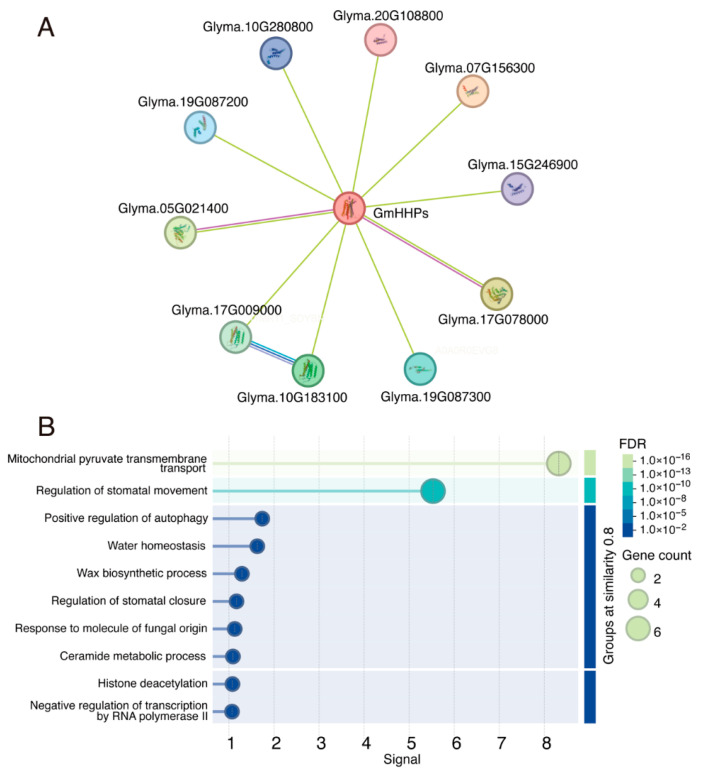
Predicted protein–protein interaction (PPI) network of GmHHPs. Protein–protein interaction (PPI) network of GmHHPs predicted using the STRING database. (**A**) GmHHPs and their putative interactors. (**B**) GO analysis of GmHHPs potential interactors enriched in biological process enrichment.

We performed GO enrichment analysis of *GmHHP* genes by using the Soybase database. These genes are enriched in GO terms related to response to salt stress (GO:0009651), response to hormone (GO:0009725), response to sucrose (GO:0009744), regulation of abscisic acid-activated signaling pathway (GO:0009788), and signaling receptor activity (GO:0038023) (Table S5). To explore the potential functions of GmHHPs, a protein–protein interaction network was constructed by querying the STRING database. In total, ten potential interacting proteins were predicted, including mitochondrial pyruvate carriers Glyma.19G087300, Glyma.19G087200, Glyma.10G280800, Glyma.20G108800, Glyma.07G156300, and Glyma.15G246900; alkaline phytoceramidases Glyma.10G183100 and Glyma.17G009000; and histone deacetylases Glyma.05G021400 and Glyma.17G078000 (Figure 10A and Table S4). GO enrichment analysis of these putative interactors revealed significant enrichment in a variety of biological processes, such as mitochondrial pyruvate transmembrane transport, positive regulation of autophagy, regulation of stomatal movement and closure, water homeostasis, and wax biosynthetic process (Figure 10B).

### 3.7. Regulation of GmHHP Genes and Their Potential Interactors by Conserved and Legume-Specific miRNA

**Figure 11:**
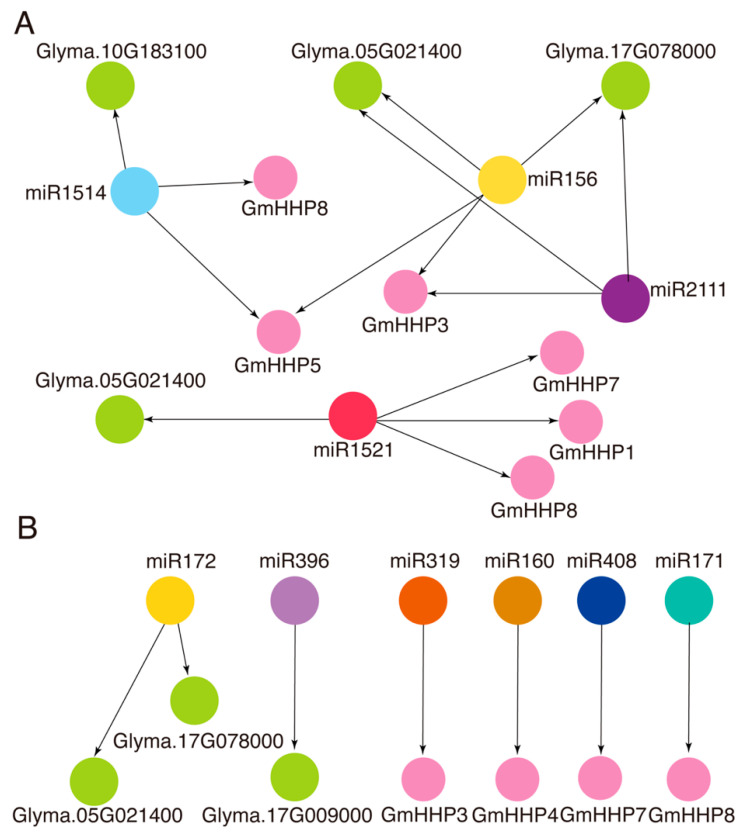
Network representation of predicted interactions between miRNAs and *GmHHP* genes and their potential interactors. (**A**) Common miRNAs regulate *GmHHP* genes and their interactors. (**B**) Conserved miRNAs predicted to regulate *GmHHP* genes and their interactors. Purple circular nodes represent *GmHHP* genes, whereas nodes of other colors represent miRNAs. Green circular nodes indicate putative protein interactors of *GmHHP* genes. Black arrows represent regulatory relationships predicted by psRNATarget.

To explore the potential post-transcriptional regulation of *GmHHP* genes and their predicted interacting proteins, miRNA–mRNA interaction network construction was performed using the psRNATarget tool. Within this regulatory network, multiple miRNAs were found to co-target both *GmHHP* genes and their interactors (Figure 11 and Table S6). For example, miR1514 was predicted to target *GmHHP5* and *GmHHP8*, as well as Glyma.10G183100. Similarly, miR156 was predicted to regulate *GmHHP3*, along with Glyma.17G078000 and Glyma.05G021400. miR1521 was found to co-target *GmHHP1*, *GmHHP7*, *GmHHP8*, and Glyma.05G021400. Notably, miR2111 was predicted to simultaneously target *GmHHP3*, *GmHHP4*, *GmHHP7*, and *GmHHP8*, along with several interacting proteins, including Glyma.05G021400, Glyma.17G078000, and Glyma.17G009000 (Figure 11A and Table S6). In addition, we also observed that several conserved miRNAs were predicted to regulate *GmHHP* genes and their interactors. For example, miRNA172 regulates Glyma.05G021400 and Glyma.17G078000, while miR319 and miR408 were predicted to regulate *GmHHP3* and *GmHHP7*, respectively (Figure 11B and Table S6).

## 4. Discussion

In plants, homologs of human progestin and adiponectin receptors have been annotated as HHPs in several species (https://phytozome-next.jgi.doe.gov/, accessed on 4 May 2025). These proteins contain a conserved Hemolysin-III–related (HlyIII) domain (Figure 1). In this study, a genome-wide analysis of soybean was conducted using HMM search, BLAST, and conserved domain validation, which identified and confirmed 10 *GmHHP* genes (Table 1 and Table S6). This gene family comprised 307 members distributed across approximately 50 distinct species. According to TAIR and PhyloGenes (https://phylogenes.arabidopsis.org/tree/PTHR20855, accessed on 4 May 2025), 5 members of this family were identified in Arabidopsis, 1 in orange, 11 in cotton, 6 in rice, 20 in wheat, and 11 in humans. This distribution indicates that the gene family is highly conserved and has undergone extensive evolutionary expansion in both plants and animals, highlighting its fundamental and conserved role in biological processes.

Recently, Zhang et al. identified homologs of human PAQRs in Arabidopsis and designated them as PAQR-like sensor (PLS) genes. Their study demonstrated that overexpression of the human adiponectin receptor AdipoRs in *A. thaliana* positively regulated plant immune responses [22]. Furthermore, in rice and soybean, *HHP* genes have been shown to enhance resistance to multiple diseases, including bacterial blight, sheath blight, and stem rot [22]. Additionally, *AtHHP* genes have been predicted to interact with KIN7, a leucine-rich repeat receptor-like kinase (LRR-RLK) that regulates stomatal closure in response to ABA and CO_2_ [22,23]. In Arabidopsis, ABA regulates the expression of *AtHHP1* and *AtHHP2*, whereas *AtHHP4/5* mRNA levels are slightly increased by ABA, and no effect is observed on *AtHHP3* [11]. The expression of stress-responsive, ABA signaling, and ABA biosynthetic genes was further elevated in the *hhp1-1* mutant under ABA or salt treatment. These findings suggest that *AtHHP1* may act as a negative regulator of ABA biosynthesis and signaling pathways in response to exogenous ABA and osmotic stress. Moreover, *AtHHP1* expression was rapidly induced within 1 h of high salinity exposure and remained elevated throughout the 24 h treatment period [12]. In Arabidopsis, salinity stress can rapidly trigger ABA accumulation, which activates ABA signaling pathways to mitigate cellular damage and regulate osmotic and ionic homeostasis in the roots [24]. These observations imply that *AtHHP1* may play diverse roles in ABA-responsive pathways. In the present study, *GmHHP* genes were also found to respond to ABA treatments (Figure 7). Based on the evolutionary analysis, *GmHHP8* and *GmHHP10* clustered closely with *AtHHP1*. Notably, both genes displayed similar expression trends under ABA treatment, showing consistent up-regulation at 10, 40, and 160 μM ABA (Figure 4 and Figure 7). These findings suggest that the functions of *GmHHP8* and *GmHHP10* in the ABA signaling pathway may differ from those of *AtHHP1*. Overall, most *GmHHP* genes were up-regulated under 10–160 μM ABA (Figure 7). *GmHHP7* and *GmHHP1* exhibited the strongest induction, with fold changes of 3.62, 8.78, and 7.96 for *GmHHP7* and 3.15, 8.18, and 7.29 for *GmHHP1* under 10, 40, and 160 μM ABA, respectively (Figure 7). However, these two genes contained relatively few ABA-responsive cis-elements in their promoters, each harboring only 2–3 such motifs. By contrast, *GmHHP8* containing the largest number of ABA-responsive *cis*-elements demonstrated lower induction levels of 4.40, 5.10, and 1.68 under 10, 40, and 160 μM ABA, respectively (Figure 7 and Table S3).

In humans, PAQR genes perform diverse functions and play essential roles in regulating energy metabolism and disease response pathways [25,26,27]. For instance, PAQR1 (AdipoR1) acts as a receptor for adiponectin, activating AMP-activated protein kinase (AMPK) to enhance fatty acid oxidation, glucose uptake, and insulin sensitivity. PAQR1 also contributes to membrane homeostasis and protects against endoplasmic reticulum (ER) stress responses [26]. Similarly, PAQR2 functions as a glucoregulatory receptor for adiponectin, primarily by activating PPAR-α signaling. Through these mechanisms, PAQR2 improves insulin sensitivity, lipid metabolism, and mitochondrial function, thereby contributing to resistance to stress and aging [25]. In Arabidopsis, *AtHHP* genes exhibit functional redundancy in PTI signaling and act as positive regulators of PTI-mediated responses and pathogen defenses. These functions included resistance to *Pseudomonas syringae* pv. *tomato DC3000*, *Botrytis cinerea*, and *Agrobacterium tumefaciens*. In rice and soybean, *AtHHP* homologs have also been implicated in defense responses against fungal pathogens [11,22]. In this study, we analyzed publicly available datasets and observed that *GmHHP* genes were differentially expressed in susceptible soybean cultivars upon nematode infection. To further investigate this, soybean roots were collected at 1, 2, and 15 dpi after inoculation with nematodes, and the expression of *GmHHP* genes was examined. The results indicated that, except for *GmHHP3*, *GmHHP5*, and *GmHHP10*, most *GmHHP* genes were down-regulated during nematode infection (Figure 8 and Figure 9). Similar trends have been reported for other membrane-associated receptor genes. For example, the receptor-like kinase GmBIR1 negatively regulates soybean immunity to SCN, with mutants exhibiting enhanced resistance [28]. Moreover, the SNARE protein GmSYP31A is up-regulated in nematode-infected roots and may contribute to vesicle-mediated defense signaling [29]. Collectively, these findings suggest that *GmHHPs* may serve as positive regulators of membrane-associated defense pathways during nematode infection.

In humans, the *PAQR* gene family interacts with multiple protein kinases, thereby influencing the AMPK signaling pathway [6,26]. In Arabidopsis, *AtHHP* genes have been shown to interact with heterotrimeric G proteins [22]. In this study, we also predicted potential interacting proteins of *GmHHPs* using the STRING database. These predicted proteins primarily included mitochondrial pyruvate carriers, alkaline phytoceramidases, and histone deacetylases. Notably, these interactors were potentially involved in biological processes such as stomatal regulation, water homeostasis, and stress signaling. Interestingly, these processes were all regulated by abscisic acid (ABA) in soybean, where ABA plays a critical role in inducing stomatal closure and maintaining water balance under osmotic stress. Under drought conditions, elevated ABA levels in soybean are associated with reduced stomatal conductance and enhanced water-use efficiency. Furthermore, cultivar-specific differences have been observed in xylem ABA sensitivity and stomatal responsiveness [30,31]. In addition, we analyzed potential miRNAs that may regulate *GmHHPs* and their interacting genes. Several miRNAs, including miR172, miR319, and miR408, were predicted to target *GmHHPs* and their associated genes (Figure 11A,B). Among these, miR172 and miR319 play critical roles in coordinating ABA-mediated developmental and defense responses during nematode challenge [32,33,34,35,36]. In Arabidopsis, miR172b is down-regulated by ABA and osmotic stress, and its overexpression increases ABA sensitivity by inducing ABA-responsive genes, highlighting its role as a regulatory switch in growth–stress balance. Similarly, soybean miR172c enhanced tolerance to water deficit and salinity but simultaneously increased ABA sensitivity, reflecting a trade-off between stress tolerance and ABA responsiveness. These findings suggest that *GmHHPs* may participate in ABA signaling and disease resistance regulation. Meanwhile, miR319 regulated JA biosynthesis by targeting TCP transcription factors and mediated RKN resistance in tomato, suggesting that interconnected hormone-miRNA defense networks may also operate in soybean SCN defense. In our soybean model, these miRNAs targeted *GmHHP* genes to modulate ABA-dependent stomatal closure, water homeostasis, and defense pathways. This integrated miRNA-*GmHHP*-ABA regulatory axis may enable soybean to fine-tune stress and immune responses during nematode infection. Future research using transgenic modulation of miRNA levels in soybean is essential to validate these regulatory links under both abiotic and biotic stress conditions.

## 5. Conclusions

This study provides comprehensive insights into the *GmHHP* gene family in soybean, highlighting its differential expression in response to ABA, MeJA, and SCN infection. The identification of potential interacting proteins and regulatory miRNAs indicates that *GmHHP* genes may be involved in ABA-mediated signaling pathways and biotic stress responses. These findings may establish a foundation for elucidating the functional roles of *GmHHPs* in hormone signaling and pathogen defense.

## Figures and Tables

**Table 1:** Physicochemical properties of GmHHPs.

Gene ID	Rename	Number of Amino Acid	Molecular Weight (Da)	Theoretical pI	Subcellular Localization
Glyma.01G194900	GmHHP1	380	43,666	8.62	Plasma Membrane
Glyma.02G207000	GmHHP2	341	37,901	8.87	Plasma Membrane
Glyma.04G046500	GmHHP3	402	46,154	8.04	Plasma Membrane
Glyma.04G156100	GmHHP4	336	38,225	9.24	Plasma Membrane
Glyma.06G047200	GmHHP5	402	46,245	8.26	Plasma Membrane
Glyma.06G223600	GmHHP6	334	37,957	8.62	Plasma Membrane
Glyma.11G046900	GmHHP7	374	42,825	8.59	Plasma Membrane
Glyma.13G161500	GmHHP8	368	42,675	8.77	Plasma Membrane
Glyma.17G070900	GmHHP9	340	37,970	8.5	Plasma Membrane
Glyma.17G109800	GmHHP10	367	42,460	8.7	Plasma Membrane

## Data Availability

The original contributions presented in the study are included in the article/Supplementary Material. Further inquiries can be directed to the corresponding author.

## Supplementary Materials

The following supporting information can be downloaded at: https://www.mdpi.com/article/10.3390/biology14091223/s1, Table S1: Soybean *HHP* gene identification information; Table S2: Comparative genomic analysis of *GmHHP* genes; Table S3: Cis-elements of *GmHHP* gene promoters; Table S4: Interacting genes of *GmHHP* genes predicted by the STRING database; Table S5: GO enrichment analysis of *GmHHP* genes. Table S6: Potential miRNAs regulate *GmHHP* genes and their interacting genes; Table S7: Primers used for qRT-PCR.
